# Extract from a Lecture on “Degeneration in Obesity”

**Published:** 1894-01

**Authors:** Clarence R. Vogel

**Affiliations:** Clinical Club, Columbus, Ohio


### EXTRACT FROM A LECTURE ON "DEGENERATION IN OBESITY."

BY DR. CLARENCE R. VOGEL, CLINICAL CLUB, COLUMBUS, OHIO.

Adipose tissue found in any part of the body, is an accumulation of fat cells held together by connective tissue more or less well supplied with blood vessels. The fats in the food are not identical to that in these tissues, as is generally supposed; by some authorities it is claimed that the fats are manufactured from the carbohydrates; that this metamorphosis takes place in some part of the body; that the amount of the fatty tissues is in no way dependent on the amount of fat in the foods; that in fatty degeneration of any of the organs, there is evidence of the formation of fat from the proteids. The fat stored up appears as fat drops or granules, and is deposited in the cell-substance which is subsequently replaced by the fat granules. This fat contains the main fat elements, olein, palmitin and stearin.

### REGARDING FATTY DEGENERATION OF THE HEART.

This organ is composed of bundles of muscular fibres bound together by connective tissue well supplied with blood vessels. In fatty degeneration the substance of the fibre cell is replaced by the fat granules, giving it the appearance of, and producing and atrophied condition of the muscular fibre cell. When this change has taken place only to a slight degree, we have the coldness of the extremeties, sighing, shortness of breath, and spells of fainting; as disintergration progresses, graver syptoms, syncope, and paralysis manifest themselves.

Many preparations have been used which would act on the oil and fat globules, either while still held by the whole corpuscles of the blood, or after they have been conveyed to the tissues involved, at the same time have no deliterious effect on the other tissues and organs of the body. The only remedy which will act in this manner has been discovered in the Phytolacca Decandra, of which preparation PHYTOLINE (Walker) is the only genuine and the only one, the efficacy of which has been demonstrated in clinical cases. From the excessive amount of oil and fat globules found on miscroscopical analysis of the urine. I am of the opinion that PHYTOLINE causes a dissolution of the fat-cells; that they are taken up by the white corpuscles and eliminated by the kidneys. That the oily matter is eliminated by the skin, is evinced by its greasy appearance, so characteristic of degeneration.

The most beneficial effects of PHYTOLINE are obtained by the patient taking 10 drops 6 times daily and large draughts of water three or four times a day and abstain from food containing carbohydrates, sugars and starches, all of which will form large constituents of ordinary fattening foods.

